# Contribution of Indian psychiatry in the development of psychiatry in Nepal

**DOI:** 10.4103/0019-5545.69216

**Published:** 2010-01

**Authors:** Tapas Kumar Aich

**Affiliations:** Universal College of Medical Sciences, Bhairahawa, Nepal

**Keywords:** India, Nepal, psychiatry

## Abstract

Psychiatric services remained virtually unknown in Nepal until 1961. The first psychiatric outpatient service was started in 1961, at Bir Hospital, Kathmandu. In 1984, the Psychiatry Department at Bir Hospital was separated and a mental hospital was created, which was later shifted to its current location at Lagankhel, Patan, in Kathmandu valley, in 1985. It is the only mental hospital in Nepal with a current bed strength of 50 beds. The new era in medical learning and teaching was ushered in Nepal with the establishment of the Institute of Medicine (IOM) under the Tribhuban University and the 400-bed Tribhuban University-Teaching Hospital (TU-Teaching Hospital), in the year 1983. BP Koirala Institute of Health Sciences (BPKIHS) at Dharan was established in 1993, as a part of the joint Indo–Nepal collaboration on developing an international standard teaching, training, and research-oriented medical institute similar to AIIMS, New Delhi. During the last one-and-half decades a number of privately run medical colleges have come up in Nepal. Outpatient and inpatient Psychiatry Departments have been established in most of these government as well as private medical institutes. At present, the postgraduate course (MD) in psychiatry has been running in two government-run institutes as well as three privately run medical colleges. Indian psychiatrists have played and are still playing significant roles in establishing as well as maintaining Psychiatry Departments, especially in the private sector medical colleges. They have also contributed to the growth of psychiatry research and postgraduate teaching in psychiatry, in Nepal.

## INTRODUCTION

### Few milestones in the development of mental health services in Nepal[1]

Psychiatric services remained virtually unknown in Nepal till 1961. Unlike other places where mental asylums first made their presence for the care of the mentally ill, mental health services started in a general hospital setting in Nepal. The first psychiatric outpatient service was started in 1961, at Bir Hospital, Kathmandu, by Dr. Bisnu Prasad Sharma, when he came back to Nepal with a DPM degree from Great Britain. A five-bed inpatient unit was established in the same hospital in 1965, which was further strengthened to 12 beds in 1971. In 1984, the Psychiatry Department at Bir Hospital was separated and a mental hospital was created, which was later shifted to its current location at Lagankhel, Patan, in Kathmandu valley, in 1985. It is the only mental hospital in Nepal, with the current strength of 50 beds. In addition to the mental hospital, in 1972, a 10-bed neuro-psychiatric unit was established in the Royal Army Hospital in Kathmandu.

### Establishment of the department of psychiatry, institute of medicine, Kathmandu[2]

A new era in medical learning and teaching was ushered in Nepal with the establishment of the Institute of Medicine (IOM) under the Tribhuban University, as also a 400-bed Tribhuban University-Teaching Hospital (TU-Teaching Hospital), in the year 1983. At the TU-Teaching Hospital, the Psychiatric Out-Patient Service was started in February 1986, followed by the addition of a 12-bed psychiatric inpatient unit in December 1987. Later, an eight-bed de-addiction unit was added to the Psychiatric Inpatient setup, thus totaling a current strength of 20 beds in the department. Clinical psychological services were offered from 1997 onward.

The Institute of Medicine was established with a view to increase the trained manpower in Nepal in the field of medicine and related fields, both at the undergraduate and postgraduate levels. Before the establishment of IOM, students from Nepal, aspiring to become doctors, had to go either to India or to the erstwhile USSR, to pursue their medical course. Postgraduate training in psychiatry was started at IOM in the year 1997, with the intake of two students for a three-year residency scheme. Subsequently, a two-year M Phil course in Clinical Psychology was started in 1998, and Bachelor of Psychiatric Nursing was started in 1999, in the same center. Indian faculties from AIIMS, New Delhi, and other PG institutes actively collaborated with their counterpart in the Department of Psychiatry, IOM, during the initial years of MD training, teaching, and examination programs.

### Establishment of the department of psychiatry, BP Koirala institute of health sciences, Dharan[3]

BP Koirala Institute of Health Sciences (BPKIHS) at Dharan was established in 1993, as a part of the joint Indo-Nepal collaboration on developing an international standard of teaching and training, and a research-oriented medical institute, similar to AIIMS, New Delhi. Outpatient psychiatric service at BPKIHS was started in 1995, after Dr. HP Jhingan and Dr. SK Khandelwal joined the institute on a short-term deputation from AIIMS, New Delhi. A 20-bed psychiatric ward was started in February 2000. The department started an MD course in Psychiatry in the same year, with the enrollment of one PG resident. Since the beginning of the PG course, visiting faculties from India and other countries formed an integral part in imparting teaching and training to PG residents in psychiatry. Some of the noted visiting faculties from India were Dr. BM Tripathy, Dr. Rakesh Lal, Dr. Indira Sharma, Dr. Avneet Sharma, Dr. RK Chadda, Dr. KMR Prasad, and Dr. Biswojit Sen. At present, every year, two to three students enrol in the MD psychiatry course.

### Establishment of the department of psychiatry in various private medical colleges in Nepal

During the last one-and-half decades a number of privately run medical colleges have come up in Nepal. With functioning teaching hospitals attached to each of these medical institutes, the In- and Outpatient Departments of Psychiatry were also established in most of them. Of late, a postgraduate course (MD) in psychiatry has been started in three private medical institutes. These institutes are, the Manipal College of Medical Sciences, Pokhara, the Universal College of Medical Sciences, Bhairahawa, and the National Medical College, Birganj.

### Contribution of Indian psychiatrists in the development of psychiatry in Nepal

As has already been stated, both the government sector medical institutes IOM, Kathmandu, and BPKIHS, Dharan, have received active assistance from eminent Indian faculty members in establishing and developing postgraduate Psychiatry Departments in both these premier teaching institutes in Nepal.

Similar cases are seen in the establishment of Psychiatry Departments in private medical colleges. Manipal College of Medical Sciences (MCMS), Pokhara, has Col. K Ramesh and Brig. PK Chakraborty on their faculty lists of the Department of Psychiatry. Both of them are eminent teachers from AFMC, Pune, and have joined the department after retirement from their service in the army. Both of them have long years of postgraduate teaching experience behind them. Brig. PK Chakraborty was the director of RINPAS, Ranchi, before joining MCMS, Pokhara as the medical director and professor of psychiatry. Thus, it came as no surprise that shortly after joining the department Brig. Chakraborty was able to start MD psychiatry in his institute, with affiliation from the Kathmandu University and the Nepal Medical Council.

The medical college in Birganj has Dr. JN Vyas, another well-known and reputed academician in the field of psychiatry in India, as head of the Department of Psychiatry. During his tenure in Nepal he helped to start a PG course in psychiatry at the Birganj Medical College. He also utilized his time in Nepal in editing and publishing the first multi-authored post-graduate psychiatry text book from India.

The present author, after completing his DPM and MD psychiatry from the Central Institute of Psychiatry (CIP), Ranchi, took the challenge of establishing a new department in another private medical college in Nepal. Present author started the outpatient service in psychiatry by January 2001, at the Universal College of Medical Sciences (UCMS), Bhairahawa. Within one year the Inpatient Department was started, with a 30-bed separate ward for psychiatry. Currently we have a 60-bed Inpatient Department (20 each for male, female, and the de-addiction center) of Psychiatry, the largest of its kind in Nepal. Our patient turnover has increased gradually over the last nine years. We attended to more than 14,000 patients at our outpatient clinic during the last one year and approximately 1200 patients were admitted and treated in our inpatient ward during the same period. These statistics are probably the highest in comparison with the performance of a Department of Psychiatry attached to a general hospital setup in Nepal. Besides the present author, the department was enriched by the presence of Dr. R Muthuswamy from Salem, India, a student of late Professor Venkoba Rao and a very popular teacher among undergraduate students here. We have started PG in psychiatry from the last academic year, with permission from the Nepal Medical Council to take one MD resident per year.

### Psychiatry research in Nepal in the new millennium

In the new millennium, research works of Nepali psychiatrists as well as Indian psychiatrists working in Nepal are published in different national and international journals. Epidemiological surveys, though in a limited scale, have been reported on psychiatric morbidity[4], alcohol dependence[5], epilepsy[6] and drugs of abuse.[7] Psychiatric morbidity studies are reported on the profiles of referred psychiatry OPD patients[8] and hospitalized medical patients[9]. There are OPD based studies[10], inpatient study reports,[9,11,12] studies on emergency psychiatry,[13] as well as study reports are available based on consultation-liaison psychiatry.[14,15,16]

Significant works have been published in relation to alcohol and drug abuse during the last one decade. There are prevalence studies on alcohol dependence,[4] psychopathology in alcohol exposed children,[16] alcohol abuse among women in Nepal,[17,18] alcohol and other substance abuse among junior doctors and medical students,[19] risk factors for drug abuse,[7] profiles of inpatient drug and alcohol abusers[12], cannabis abusers[20], injection drug abusers[21,22] and family burden in opioid dependence patients.[23]

Published literature are available on diverse topics like co-morbidity in psychiatric illnesses,[24,25] quality of life,[26] Nepali adaptation of international guidelines and rating scales,[27,28] headache[29], HIV[30] and depression during pregnancy[31].

The Indian Psychiatric Society (IPS) always encouraged research article presentations by psychiatrists from Nepal during its annual national conferences (ANCIPS).[4,10] The Indian Journal of Psychiatry (IJP) also encouraged publications of research articles received from Nepal.[14,32,33]

Two research articles of the present author were published in IJP, while working in Nepal, in the year 2004 and 2005, although the study was carried out in India.[24,25] Two more articles of the present author, based on the research study done in Nepal, have been accepted for publication recently.[11,22]

## EPILOGUE

This is a humble effort to outline the growth of modern psychiatry in Nepal and the contribution from Indian psychiatrists in its development. Some important references might have been missed, in an attempt to write this article within a very short time frame.

This article would remain incomplete if I did not mention the name of Professor Mahendra Kumar Nepal, an alumnus of the Agra Medical College and MD psychiatry from AIIMS, New Delhi. He was the key person in establishing the Department of Psychiatry in IOM, Kathmandu. A person with vision, great leadership quality, and a tireless worker, he was able to influence the decision-makers in the Tribhuban University and Nepal Medical Council to introduce a separate clinical examination for final MBBS students, which included presenting a long case before the examiner and appearing for a viva in psychiatry and related subjects. Undergraduate psychiatry teaching and learning in the Institute of Medicine, Kathmandu, includes 40 hours of theory classes and 160 hours of clinical exposure for the final year MBBS students. This is where, probably, psychiatry in Nepal outscored India in terms of the undergraduate psychiatry curriculum followed and the pattern of examination conducted.

Professor Mahendra Kumar Nepal was the main driving force behind the start of the Nepalese Journal of Psychiatry in the year 1999, although its publication was stopped after a few years. It was upon his personal effort that Nepal successfully conducted the second SAARC Psychiatric Conference in Kathmandu, in November 2006. ‘Brain drain’ in psychiatry also affected Nepal, with at least three psychiatrists leaving the country in search of greener pastures abroad. Probably the bigger loss to psychiatry in Nepal was when Dr. MK Nepal also decided to join the Australian Government service a few years back! Hopefully newer, younger, and competent leaders like Professor VD Sharma of IOM, Kathmandu and Professor PM Shyangwa of BPKIHS, Dharan, will ably fill up the vacant place left by Professor MK Nepal and will take psychiatry in Nepal to newer heights in the coming decade.
